# Identification of chemical and physical key water quality drivers in the urban Grunewald Chain of Lakes, Berlin

**DOI:** 10.1038/s41598-026-53251-7

**Published:** 2026-05-16

**Authors:** Christina F. Radtke, Nick Heinemann, Arne Höring, Kai Schröter

**Affiliations:** Leichtweiss Institute for Hydraulic Engineering and Water Resources, Division of Hydrology and River Basin Management, Technische Universität Braunschweig, Braunschweig, Germany

**Keywords:** Connected water bodies, Nutrient limitation, Urban lakes, Monitoring, Water quality, Ecology, Ecology, Environmental sciences, Hydrology, Limnology, Water resources

## Abstract

Aquatic ecosystems are threatened by high nutrient loads. Particularly urban lakes that are used as storm water reservoirs are polluted by phosphorus, nitrogen and other pollutants. Improving the water quality of urban lakes is both a benefit for the ecosystem, and for the socio-ecological value of the waterbody. This study investigates the Grunewald chain of lakes in Berlin, Germany which is threatened by high nutrient loads from surrounding urban areas. To date, measures to improve the water quality failed to achieve a resilient, long-term balanced and stable aquatic ecosystem. Connected lakes pose major challenges for water management due to their interactions. To better understand the exchange of nutrients in the Grunewald chain of lakes, a monitoring campaign and data analysis were conducted, with monthly water samples over a period of 13 months at 17 sampling stations, focusing on the inlets, outlets and connections of the lakes. This study reveals the relevance of temperature, volume ratio, depth and phosphorus concentrations affecting the nutrient limitation of the lakes and how water quality of the lakes are affected by each other. The study gives insights to cascading effects on nutrient accumulation along a chain of lakes, providing guidance for further management practices.

## Introduction

Urban lakes are rewarded for their multifunctional role as blue-green oasis within cities. They are used for recreational activities of inhabitants and tourists, such as bathing and to experience nature. For the urbanized area they provide a cooling effect on the surrounding, and urban lakes can be used as flood protection areas as well as storm water reservoirs of surrounding traffic areas. Within cities, lakes offer habitat structures for aquatic species like fishes, mammals and plants and by this they serve as stepping stone biotopes^1,2^. Even though this multifunctional role shows the socio-ecological value of the lakes, they are threatened by high loads of nutrients and contaminants that are transported in the lakes via runoff from the surrounding urban areas^3^. The anthropogenic impacts on the water quality of lakes cause eutrophication^4^ and fish disease^5^ as well as odor due to sulphureous gases^6^. Special cases are connected water bodies where cascading effects of decreasing water quality^7–11^, but also a positive effect of water quality management measures have been found^12^. In the study of Kuriata-Potasznik et al.^8^ lakes that are connected with streams were investigated as connected water bodies. They found that lakes act as nutrient traps that accumulated the nutrient loading that enter the lake via the stream, which results in a decreasing water quality along the flow path from the river to the lake. Instead, Hilt et al.^12^ showed with a model of a chain of lakes that the water quality of connected water bodies can be improved due to an improvement of the water quality upstream. It is important to better understand how connected water bodies interact with each other and how water quality of chain of lakes can be improved for all and individual lakes^13^. This study focuses on the Grunewald chain of lakes in Berlin, Germany. The chain of lakes consists of ten lakes that are connected with each other via pumps, canals, streams through wetlands or directly via a short stream (2–5 m). The lakes are imbedded in channels from the Weichsel Glaciation and ground moraine formation. In the past, these lakes suffered from high nutrient loads coming from the Spree River. Agricultural areas in the Spree catchment led to high nutrient concentrations in the Spree River that were transported further into the Fennsee which was previously the most upstream lake of the chain of lakes. With the reverse of the flow direction in 1981, the lake Fennsee is now the most downstream lake of the chain of lakes. Due to the high nutrient loads, measures have been taken to improve the water quality. The flow direction has been reversed with pumps, including a surface water treatment facility where phosphorus concentrations are reduced before water flows from the Havel River to the Grunewald chain of lakes. Different filter systems for water flowing from the streets to the lakes were implemented aiming to reduce the nutrient load. But still the Grunewald chain of lakes suffers from high loads of nutrients and it has not been investigated how the connected lakes interact with each other or affect the nutrient situation of each other. To describe the effect of nutrient loading in aquatic systems on biodiversity, the TN: TP ratio can be calculated using the concentrations of total nitrogen (TN) and total phosphorus (TP). The TN: TP ratio gives information on which nutrient should be limited to reduce the algae growth in the water body. Most studies^7,14–18^ investigated large amounts of different lakes all over the world, where they investigated the nutrient limitation and the effects on the TN: TP ratio. Some studies found that often it is not only one of the nutrients that has to be limited, instead a Co-limitation of both nutrients is recommended to improve the water quality of lakes^19–21^. So far there is no study that focused specifically on urban lakes that are connected and how the ratio of total nitrogen and total phosphorus varies along the flow path. Using the Boruta analysis, McCullough et al.^16^ showed in a study about more than 3000 lakes in the United States that the relationship between nutrients and chlorophyll-a are most relevant in the context of nutrient limitation in lakes and that nutrient limitation of lakes is related to a combination of watershed characteristics, regional scales and the environmental conditions of the lakes. Aiming to understand which factors affect the urban lakes of the Grunewald chain of lakes in Berlin, we used the Boruta analysis to investigate how lake characteristics, physical and chemical parameters are related to the TN: TP ratio.

The complex system of the Grunewald chain of lakes consists of ten lakes and several inflows from storm water runoff that originate from eleven catchments. To better understand the complex system, a monitoring campaign was conducted over 13 months, focusing on the inlets, outlets and direct connections of the lakes. The aim was to investigate [i] the rate of nutrient transport along the lakes and if this affects downstream lakes and [ii] to unravel important drivers for nutrient limitation in the Grunewald chain of lakes, using the Boruta analysis. First, the materials and methods are presented with the study area, monitoring campaign, laboratory analysis and data analysis. Results follow thereafter with an evaluation of the physical and chemical parameters and nutrient concentration in the chain of lakes, obtained during the monitoring campaign. Hereinafter, the parameter importance on nutrient limitation in urban lakes is presented. In the discussion, the pollution of the lakes is discussed as well as the shift of the limiting nutrient, followed by the discussion about the parameters affecting the TN: TP ratio. The manuscript concludes with an assessment of the monitoring approach and the data analyses methods and provides recommendations for the water quality management of the chain of lakes.

## Materials and methods

### Study area

**Fig. 1:**
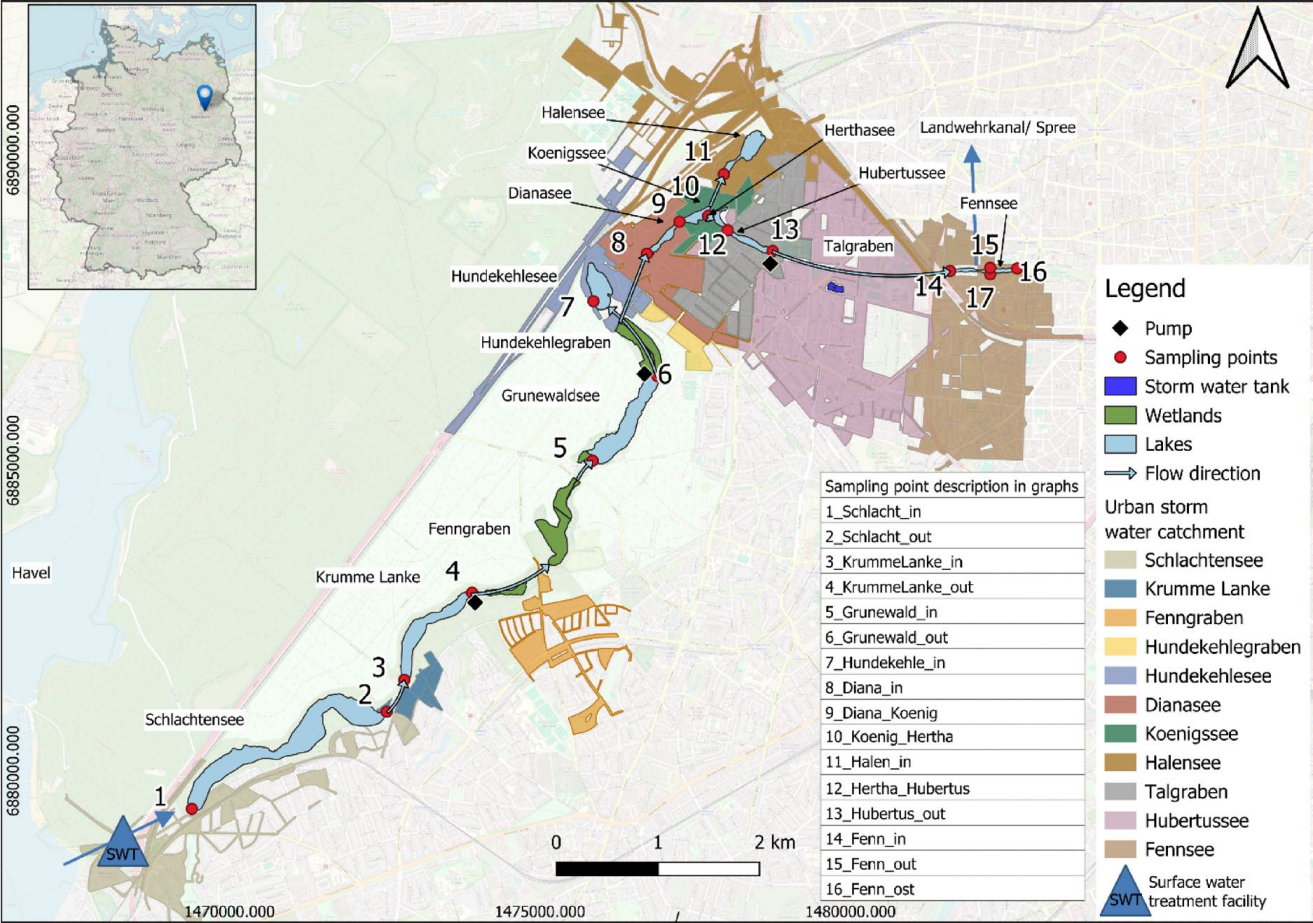
Grunewald chain of lakes in Berlin, Germany with the urban storm water catchments of each lake and wetlands that contribute to the whole chain of lakes. In the storm water catchments^23^, runoff is collected in canals which transport the polluted water to the lakes. A special case are the catchments of Talgraben and Hubertussee: the grey urban storm water catchment drains into the Talgraben directly via canals; the pink catchment drains into canals that transport water into the Hubertussee close to the sampling point 13. Monthly sampling points of water quality measurements are shown as red dots, black diamonds are pumping stations operated by the Berliner Wasserbetriebe. Blue arrows indicate where water enters and leaves the Grunewald chain of lakes. The map was created with QGIS^24^ version 3.34.3 using OpenStreetMap Standard^25^ as background map.

The Grunewald chain of lakes is located in the southwest of Berlin, Germany, within a temperate climate zone. The average air temperature from 2020 to 2025 is 10.85 °C and the average precipitation per year is 477 mm during the same period at the weather station Berlin-Dahlem^22^. In July 2025 several heavy rainfall events took place with precipitation totals of 111 mm measured at the weather station Berlin-Dahlem. The monitoring in July 2025 took place right after a heavy rain fall event with more than 20 mm of rain on one day^22^. The lake chain is embedded in gyttjas and peat, surrounded by outwash plain (Sander) from the Weichsel Glaciation and ground moraine formation. Ten lakes are connected via pumps, channels or directly with each other (Fig. 1). The natural flow direction has been reversed in 1981 to improve the water quality. Nowadays, phosphorus reduced water (< 10 µg/l total phosphorus) from the river Havel that is purified at the surface water treatment (SWT) plant Beelitzhof enters the lake Schlachtensee which is connected with the lake Krumme Lanke via a creek. Pumps that are operated by the Berliner Wasserbetriebe (Berlin water supply company) were installed in 1981 to transport the water against the topographic gradient. From the lake Krumme Lanke water flows through a creek within a wetland and is transported with a pump with a capacity up to 450 m³/h upwards to the lake Grunewaldsee. The outflow of the lake Grunewaldsee enters another creek that flows through a wetland. A pump with a capacity up to 350 m³/h transports water at the end of the creek to the lake Hundekehlesee as well as to the lake Dianasee. The lakes Dianasee, Königssee, Herthasee and Hubertussee are directly connected with each other and via a channel the lake Halensee is connected with the Königssee. Another pump with a capacity up to 200 m³/h transports water from the lake Hubertussee to the channel Talgraben which enters the lake Fennsee. From the lake Fennsee water flows over an overflow threshold and then through a channel to the river Spree. A storm water tank is located between the lakes Hubertussee and Fennsee. Water that is collected in the storm water tank enters the Hubertussee close to the pumping station at the outlet of the lake Hubertussee (Fig. 1).

Schlachtensee, Krumme Lanke and Grunewaldsee have the largest surface area and the highest volume (Table 1). These lakes are natural and they are located in a forested area and they do not receive rainwater inflows from the urban areas anymore. Instead, the lakes Dianasee, Königssee, Hubertussee, Fennsee, Halensee and Hundekehlesee are surrounded by urban areas and these lakes are mainly used as rain water collectors from the motorway and the surrounding traffic areas. The lakes are used as local recreational area of inhabitants and tourists and they offer aquatic habitats for plants and animals. Due to runoff from traffic areas that enters the lakes and transports nutrients, salts and harmful substances the ecosystem is polluted causing eutrophication, odor nuisance and fish disease in the lakes. The volume ratio, which describes the relationship between the lake volume and the connected catchment areas is highest for the Hubertussee and the Fennsee. The highest amount of storm water runoff flows into the Talgraben, which connects the lakes Hubertussee and Fennsee. Characteristics of all lakes are listed in Table 1.

**Table 1:** Characteristics of the lakes from upstream to downstream^26^.

Lakes	Volume	Extent	Mean depth	Max. depth	Area	Urban runoff Catchment	Volume ratio
	m^3^	m	m	m	ha	ha	m^2^/m^3^
Schlachtensee	1,938,747	5506	4.66	8.91	41.56	0.00	0.00
Krumme Lanke	543,328	2578	3.88	6.77	14.02	0.00	0.00
Grunewaldsee	504,574	2617	2.92	6.45	17.27	0.00	0.00
Hundekehlesee	210,464	1204	3.01	6.69	7.00	40.50	1.92
Dianasee	41,644	1115	1.73	4.50	2.41	73.00	17.53
Königssee	40,974	779	1.92	3.88	2.13	24.78	6.05
Halensee	179,363	1422	3.21	7.81	5.59	68.00	3.79
Herthasee	15,974	820	1.39	3.11	1.15	0.00	0.00
Hubertussee	42,143	1066	1.79	3.90	2.36	311.00	73.80
Fennsee	41,153	1409	1.91	4.29	2.15	169.00	41.07

The nutrient loads of the storm water from surrounding areas are known to affect the ecosystem. Several measures^27–29^ were taken to improve the water quality, but without a long-term success. In previous monitoring programs, the water quality of individual lakes was analyzed. The lakes Schlachtensee, Krumme Lanke and Halensee have beaches and are known as bathing lakes. Therefore, a continuous monitoring program from April to September each year is conducted by the state agency according to the Bathing Water Directive^30^. In 2016 several lakes were monitored monthly from April to September: Dianasee, Königssee, Herthasee, Hubertussee and Fennsee. In 2017 another monthly monitoring campaign was conducted from March to October at the lakes Grunewaldsee, Dianasee at two locations, Hundekehlesee and Hubertussee at two locations. The monitoring campaigns in 2016 and 2017 identified the trophic state of the lakes (Table 2)^31^. Since 2021 the lakes Halensee and Fennsee are monitored monthly (with some gaps) commissioned by the district office Charlottenburg-Wilmersdorf^32^. So far, there has not been a monitoring campaign that covered all ten lakes over a full year with measurements on the same day. Therefore, information is missing about how the lakes affect each other and if a cascading effect on water quality takes place in the chain of lakes as highlighted in other studies^7–12^.

**Table 2:** Trophic state of the lakes^31^.

Lakes	Year	Trophic index	Trophic state
Grunewaldsee	2017	2.12	Mesotrophic
Hundekehlesee	2017	3.15	Eutrophic
Dianasee	2017	3.23	Eutrophic
Koenigssee	2016	3.23	Eutrophic
Halensee	2017	3.30	Eutrophic
Herthasee	2016	3.45	Eutrophic
Hubertussee	2017	3.74	Hypertrophic
Fennsee	2016	4.13	Hypertrophic

### Monitoring campaign

To better understand the nutrient load that is transported from one lake to another, a monitoring campaign was conducted starting in July 2024 and ending in July 2025. A monthly sampling of the surface water at the inlets, outlets and at the direct connections of the lakes was carried out which resulted in 17 sampling locations (Fig. 1) and 204 water samples in total. The station 16 was only measured once due to heavy vegetation at the shoreline and difficulties to reach the water phase. Station 17 was sampled since October 2024. In the field the parameters water temperature, pH-value, electrical conductivity and oxygen concentration were measured with the device WTW Multi 3630 IDS. Chlorophyll-a, water temperature, electrical conductivity and salinity were measured with the multiparameter probe YSI 6150. Water samples were held in 250 ml amber glass bottles and stored at 4 °C until analysis on the following day in the laboratory.

### Laboratory analysis

Water samples were analyzed regarding concentrations of total phosphorus, ortho-phosphate and total nitrogen. A doubled analysis was conducted to reduce the uncertainty of the results. In total there were 455 analyses of total phosphorus, 443 analyses of ortho-phosphate and 398 analyses of total nitrogen. In July and August 2024 not all water samples were measured concerning the total nitrogen concentration because of a lack of reagents.

To analyze the water samples in the photometer, water samples were prepared with test kits. Total phosphorus and total nitrogen were analyzed using unfiltered water samples and the Crack Set 10 and Crack Set 20 from the company Supelco, respectively. For total nitrogen DIN EN ISO 11905-1 is applied while for total phosphorus DIN 38405-9 is applied. For ortho-phosphate DIN EN ISO 6878 is applied. In July 2024 the photometer Spectroquant NOVA 60 of the company Merck was used and since August 2024 the photometer Spectroquant Prove 100 plus was used for further analyses.

### Data analysis

Data that was obtained in the field and in the laboratory was evaluated and analyzed using Excel from the package Microsoft Office 2021^33^ and R version 4.4.3^34^ and RStudio^35^. With the empirical variance the doubled analysis of the nutrients was tested against the uncertainty of the data. Concentrations that obtained an empirical variance higher that 0.1 were repeated. Due to the change of the photometer and due to old and contaminated cuvettes the variance of total phosphate was 3.4 in July 2024 and 1.18 on August 2024. From September 2024 to July 2025 the variance for total phosphorus was 0.0051. In July 2024 the variance of ortho-phosphate samples was 0.0056 and from August 2024 to July 2025 the variance was 0.000038. For total nitrogen the variance in July 2024 was 0.055 and from August 2024 to July 2025 the variance was 0.014. In July and August 2024, the variance of the total phosphorus analyses was 3.4 and 1.18, respectively. Therefore, for all duplicate analyses the lower concentration values were chosen for further data analyses to avoid biases. In most cases the difference between the analyses were below the threshold of 0.1 e.g., 0.0051 for total phosphorus for analyses from September 2024 to July 2025.

To analyze which nutrient is limiting the algae growth, the TN: TP ratio, also known as Redfield ratio^36^ is investigated based on the nutrient mass for each sampling date. Following Paerl et al.^21^ the TN: TP ratio based on nutrient concentrations can be categorized in P-limited (TN: TP > 23), N-limited (TN: TP < 9) or Co-limited (TN: TP > 9, TN: TP < 23) and it provides the information which nutrient should be reduced to improve the water quality of a lake^21,37–41^. Lakes that are P-limited have low phosphorus concentrations and measures should be taken to keep the phosphorus concentration low. In N-limited lakes, nitrogen concentrations are low and measures should avoid an increase of nitrogen in the ecosystem. Co-limitation occurs predominantly in eutrophic lakes when both nutrients, nitrogen and phosphorus are available in high concentrations. In the range of Co-limitation, P- and N-limitation alternate quickly due to rapid changing nutrient concentrations e.g., after high release of phosphorus from the sediment, the P-limitation can quickly change to N-limitation. The data that was obtained during the monitoring campaign are located at the inlet, outlet and the direct connections of the lakes. Usually the TN: TP ratio is calculated for concentrations that were measured at the middle of the lakes. With the data obtained in the monitoring campaign, the local conditions of the inflowing and outflowing water can be described. By measuring the nutrient concentrations at the inlet and the outlet of the lakes, the nutrient transport along the chain of lakes can be assessed compared to measurements in the middle of a lake.

To analyze the influences of different lake specific characteristics such as lake depth or volume ratio and the obtained parameters such as chlorophyll-a concentrations, physical and chemical parameters or nutrient concentrations on the TN: TP ratio a Boruta analysis is conducted. With the Boruta analysis the importance of parameters that affect the target variable can be investigated^42^. Based on random forest algorithm^43^ and the idea of Stoppiglia et al.^44^, the Boruta feature selection was developed. Stoppiglia et al.^44^ introduced the approach to identify significant input parameters from a data set with many dependent variables, by using a duplicate random variable which is also called a shadow variable. The shadow variables are shuffled copies of the original data set, which are added to the original data set for further analyses of several random forest classifier runs. Z scores are computed during the random forest algorithm and the maximum Z score among shadow variables is then assigned to be the threshold for determining the importance of the variables in the original data set. When the importance of a variable is higher than the maximum of the shadow, the parameter is evaluated as being important for the change of the target variable, otherwise the parameter is rejected. Due to a high number of random forest models, this method gives robust results. Here, 645 iterations of random forest models were conducted within the Boruta algorithm. The Boruta analysis was conducted using the R package *Boruta*^45^. By using the TN: TP ratio as target, the parameters of the monitoring campaign and the lake characteristics were investigated concerning their importance for the TN: TP ratio.

## Results

### Physical and chemical parameters

During the monitoring campaign at the Grunewald chain of lakes, physical and chemical parameters were measured. Table 3 gives an overview of the average values for each lake considering the measurements at the inflow and at the outflow of the lake. Along the lake chain electrical conductivity and salinity are decreasing. Increasing concentrations of chlorophyll-a, total phosphorus and ortho-phosphate can be found from Schlachtensee to Fennsee. Total nitrogen is highest in the Schlachtensee and varies between 1.03 and 1.36 mg N/l along the other lakes with an increasing trend in the directly connected lakes from Koenigssee to Fennsee, considering the average values of the monitoring campaign. The TN: TP ratio is higher in the lakes from Schlachtensee to Grunewaldsee with values ranging from 20 to 27, showing a clear phosphorus limitation at lakes that are located within the forested area. At the other lakes, that are surrounded by urban area and have high ratios between the volume of the lake and the area of urban runoff, average TN: TP is ranging from 8 to 19, showing a co-limitation of both nutrients, nitrogen and phosphorus.

**Table 3:** Average values of the measured parameters during the monitoring campaign with O_2_ = Oxygen concentration, EC = Electrical Conductivity, T = Temperature, pH = pH-value, Chl-a = Chlorophyll-a concentration, Salt = Salinity, TN = Total Nitrogen concentration.

Lakes	O_2_ [mg/l]	EC [µS/cm]	T [°C]	pH [-]	Chl-a [mg/l]	Salt [mg/l]	TN [mg N/l]	TP [mg P/l]	PO4P [mg P/l]	TN: TP ratio
Schlachtensee	9.01	810.94	13.96	7.90	4.13	0.40	1.44	0.05	0.03	26.95
Krumme Lanke	9.09	796.04	13.62	8.05	4.22	0.40	1.20	0.06	0.03	20.39
Grunewaldsee	9.45	769.12	13.44	7.99	5.45	0.38	1.06	0.04	0.02	24.38
Hundekehlesee	8.20	739.38	13.80	7.89	7.57	0.37	1.13	0.06	0.02	19.70
Dianasee	8.21	726.24	13.82	7.83	8.74	0.36	1.09	0.08	0.03	12.87
Koenigssee	8.74	727.50	13.79	7.90	11.85	0.36	1.03	0.06	0.02	16.74
Halensee	7.19	718.62	13.39	7.84	10.68	0.37	1.36	0.08	0.03	16.12
Herthasee	8.54	711.27	13.44	7.79	13.35	0.35	1.05	0.13	0.02	8.36
Hubertussee	6.53	629.80	13.40	7.57	14.05	0.31	1.13	0.10	0.04	11.82
Fennsee	7.14	652.33	12.54	7.50	13.69	0.33	1.36	0.13	0.07	10.078

**Fig. 2:**
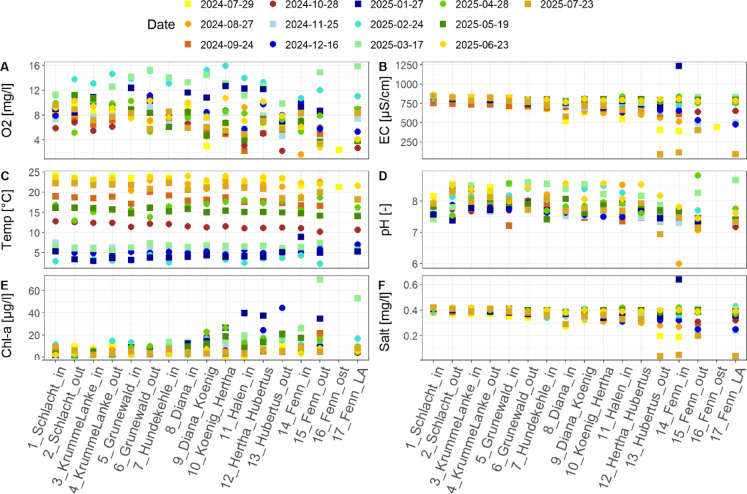
Physical and chemical parameters from field measurements with oxygen concentration (O2 [mg/l]) in panel A, electrical conductivity (EC [µS/cm]) in panel B, water temperature (Temp [°C]) in panel C, pH-value (pH [-]) in panel D, chlorophyll-a concentration (Chl-a [µg/l]) in panel E and salinity (Salt [mg/l]) in panel F. Sampling stations are on the x-axis in the flow direction of the water. Yellow colors show measurements in summer, orange to red colors show measurements in autumn, blue colors show measurements in winter and green colors show measurements in spring.

In Fig. 2 the physical and chemical parameters obtained during the monitoring campaign are shown. It is noticeable that the oxygen concentration is reduced along the flow path through the lakes. At some dates the oxygen concentration was below 3 mg/l at the stations 13 to 17 which is a harmful condition for fishes. The lowest oxygen concentrations were mainly found in summer and autumn. Electrical conductivity varies between 500 and 800 µS/cm for the stations 1 to 11 whereas the electrical conductivity varies between 90 and 800 µS/cm at the following stations 12 to 17 in the lakes Hubertussee and Fennsee. Similar observations can be found for the salinity of the lakes. The highest electrical conductivity (1233 µS/cm) and salinity (0.64 mg/l) were found at station 14. At the lake Fennsee (Stations 14–17), water temperature is more likely to be higher during winter and lower during summer compared to other lakes and stations. For all lakes the pH-value varies between 7 and 9 with one exception in August 2024 at station 14 with a pH-value around 6. From Schlachtensee, station 1 to the station 7, Hundekehlesee chlorophyll-a ranges from 1 to 20 mg/l. Especially at lake station 8 (Dianasee), the concentration of chlorophyll-a starts to increase above 20 mg/l with the following lakes, while the highest chlorophyll-a concentration with 70.1 mg/l is found in the lake Fennsee in spring 2025.

### Nutrients in the chain of lakes

**Fig. 3:**
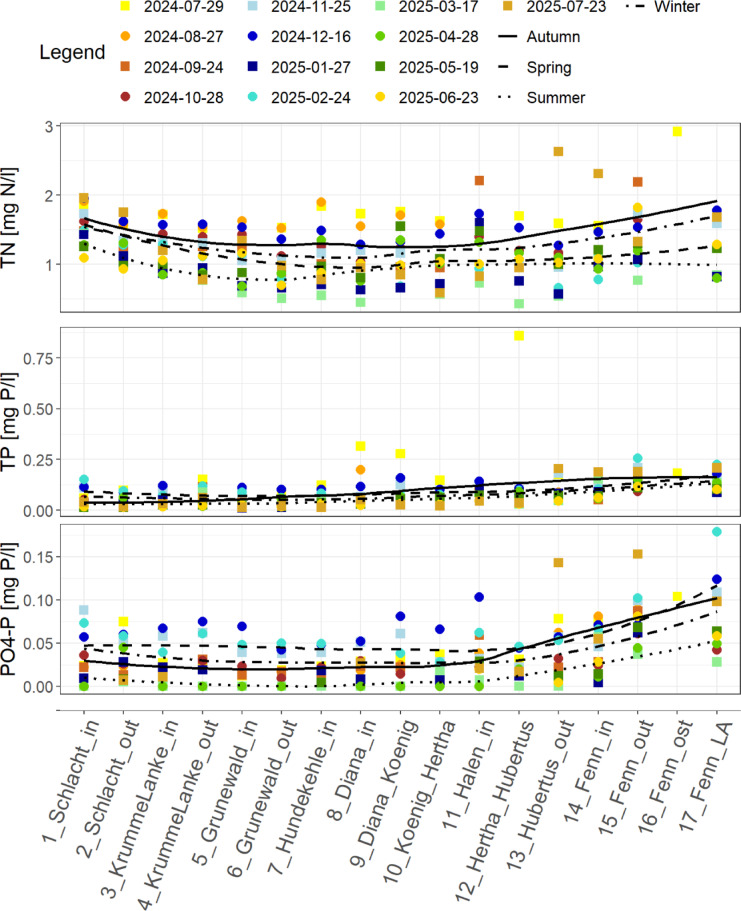
Nutrient concentrations from field measurements with total nitrogen (TN [mg N/l]) in the top panel, total phosphorus (TP [mg N/l]) in the middle panel and ortho-phosphate (PO4-P [mg P/l]) in the bottom panel. Sampling stations are on the x-axis in the flow direction of the water. Yellow colors show measurements in summer, orange to red colors show measurements in autumn, blue colors show measurements in winter and green colors show measurements in spring. The black lines show the distribution over the seasons along the chain of lakes.

The nutrient concentrations in Fig. 3 visualize the contamination at the inlets, outlets and the connections of the lakes during the monitoring campaign. Total nitrogen concentrations are higher at station 1 and decrease along the lake Schlachtensee to a lower concentration in the outlet of the lake at station 2. In the lakes Krumme Lanke and Grunewaldsee total nitrogen concentrations decrease and stay between 0.4 mg N/l and 2 mg N/l over the whole monitoring campaign for the following lakes Dianasee (stations 8 and 9), Hundekehlesee (station 7), Königssee (stations 9 and 10) and Herthasee (stations 10 and 12) as well. Total nitrogen concentrations vary between 0.6 and 2.5 mg N/l in lake Halensee (station 11). In lake Fennsee (stations 14 to 17) the total nitrogen concentration is highest and varies between 0.7 and 3 mg N/l. In summer total nitrogen is highest for all measured stations compared to the other seasons. In spring lowest total nitrogen concentrations are found for all stations (Fig. 3). Total phosphorus concentrations are below 0.25 mg P/l from lakes Schlachtensee to Hundekehlesee (stations 1 to 7) in contrast to the whole chain of lakes. The phosphorus concentrations increase up to 0.4 mg P/l in lake Dianasee (stations 8 and 9). At sampling stations of the lake Fennsee (stations 14 to 17) total phosphorus concentrations are between 0.1 and 0.3 mg P/l. Along the chain of lakes, the concentrations are highest in winter for stations 1 to 7 and from stations 8 to 16 the total phosphorus concentrations are highest during summer. For all stations the lowest total phosphorus concentrations are found in spring (Fig. 3). A similar seasonal pattern is found for phosphate. The phosphate concentrations are highest during winter for stations 1 to 11 and from stations 12 to 16 the highest phosphate concentrations are found during summer. In spring the lowest phosphate concentrations are found for all stations (Fig. 3). The concentrations of ortho-phosphate vary from lake Schlachtensee (stations 1 and 2) along the lakes to lake Königssee (stations 9 and 10) between 0 and 0.1 mg P/l, while ortho-phosphate increases during winter in lake Halensee (station 11). At sampling stations from lake Fennsee (stations 14 to 17) ortho-phosphate varies between 0.05 and 0.2 mg P/l which are the highest variations of the whole chain of lakes.

**Fig. 4:**
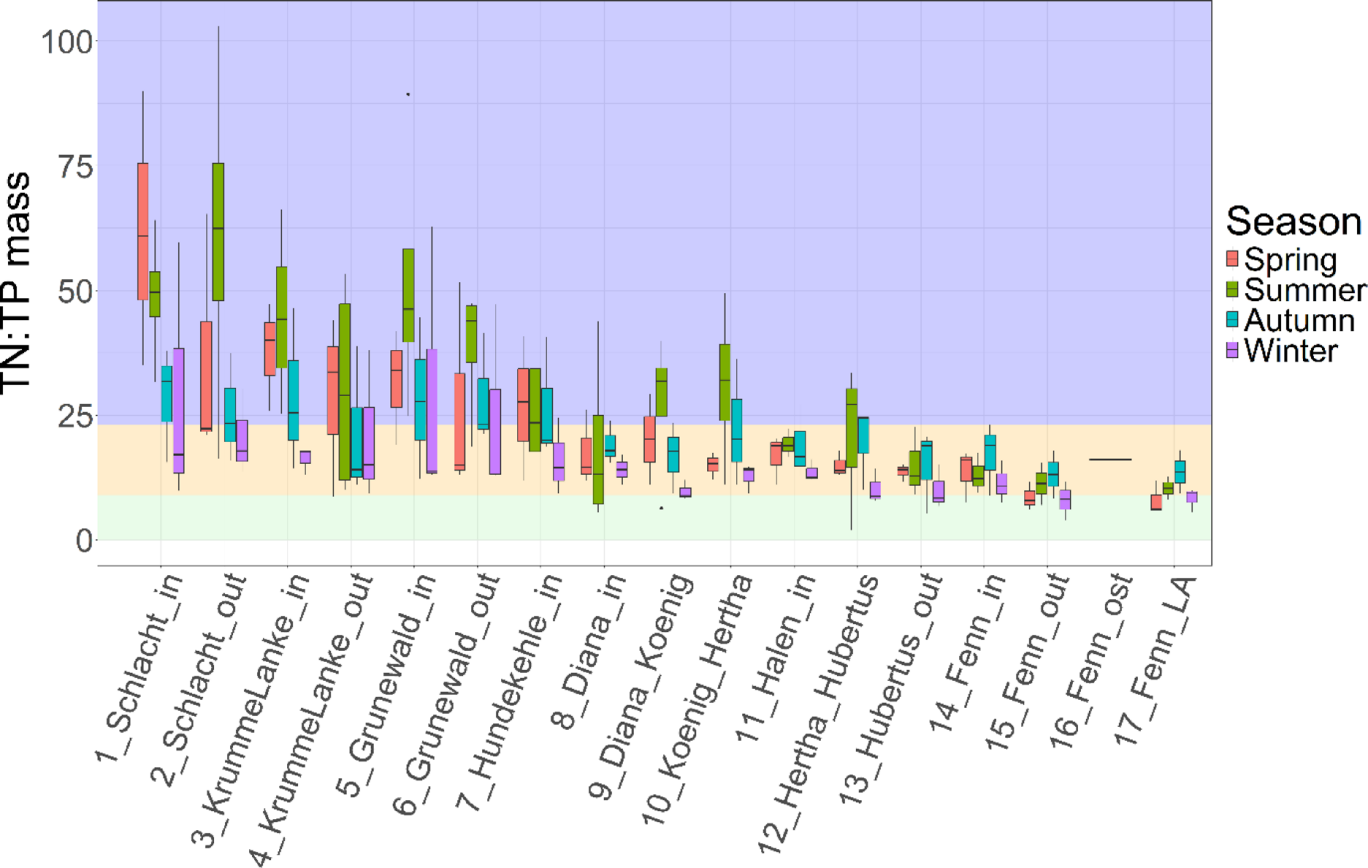
TN: TP ratio with sampling stations in flow direction along the x-axis and ranges for P-, N- and Co-limitation (P-limitation = TN: TP > 23 in blue, N-limitation = TN: TP < 9 in green, Co-limitation = TN: TP > 9 and TN: TP < 23 in orange) with boxplots for different seasons during the monitoring campaign from July 2024 to July 2025.

Using the TN: TP ratio in Fig. 4 the limiting nutrient can be estimated from the data. The lakes Schlachtensee (stations 1 and 2), Krumme Lanke (stations 3 and 4) and Grunewaldsee (stations 5 and 6) are mainly P-limited during spring, summer and autumn and they are Co-limited during winter. Co-limitation also occurs during summer and autumn at lake Krumme Lanke (stations 3 and 4) and during spring at lake Grunewaldsee (stations 5 and 6). Most of the time lakes from Dianasee until Fennsee (stations 8 to 17) are Co-limited with some exceptions during summer at the connection between the lakes Dianasee (stations 8 and 9) and Königssee (stations 9 and 10) as well as the connection between Königssee and Herthasee (station 10) where the measurements show a P-limitation. In the lake Fennsee a N-limitation can be found at the outlet of the lake and at station 17, mainly during spring and winter.

Focusing on the inlets, outlets and the connections of the lakes the TN: TP ratio is higher at the inlets of the lakes Schlachtensee (station 1), Krumme Lanke (station 3) and Grunewaldsee (station 5) compared to their TN: TP ratio at the outlets (stations 4 and 6), except for the outlet of the Schlachtensee (station 2) in summer where a higher TN: TP ratio is found compared to the inlet (station 1). Instead, at lake Dianasee the TN: TP ratio is higher at the connection to the Königssee (station 9) compared to its inlet (station 8) TN: TP ratio. At the connections between the lakes Herthasee, Hubertussee and Fennsee (stations 12 and 13) the TN: TP ratio is higher at the inflow compared to the outflow of the lakes. Due to lake intern retention of nitrogen, the lowered nitrogen concentrations in the lakes Schlachtensee, Krumme Lanke and Grunewaldsee cause an increase of the TN: TP ratio at the outlet of the lakes. In the lakes Dianasee until Fennsee the inflowing rain water expected from measured rainfall at station Berlin-Dahlem that flows from traffic areas to the lakes dominate the shift of the TN: TP ratio in the connections of the lakes, which is also visible in Fig. 3 where the seasonal curves of total phosphorus and phosphate are increasing starting from the Dianasee. The increasing concentrations of total phosphorus and phosphate from lake Dianasee to lake Hubertussee (stations 8 to 13) show the cascading effect on the TN: TP ratio of the lakes which is lower at the outlets compared to the inlets. The strong increase of total phosphorus concentrations in common with total nitrogen concentrations that have just a small increase compared to the phosphorus concentrations, show how water that is loaded with nutrients is transported from one lake to the other.

### Parameter importance on nutrient limitation in urban lakes

**Fig. 5:**
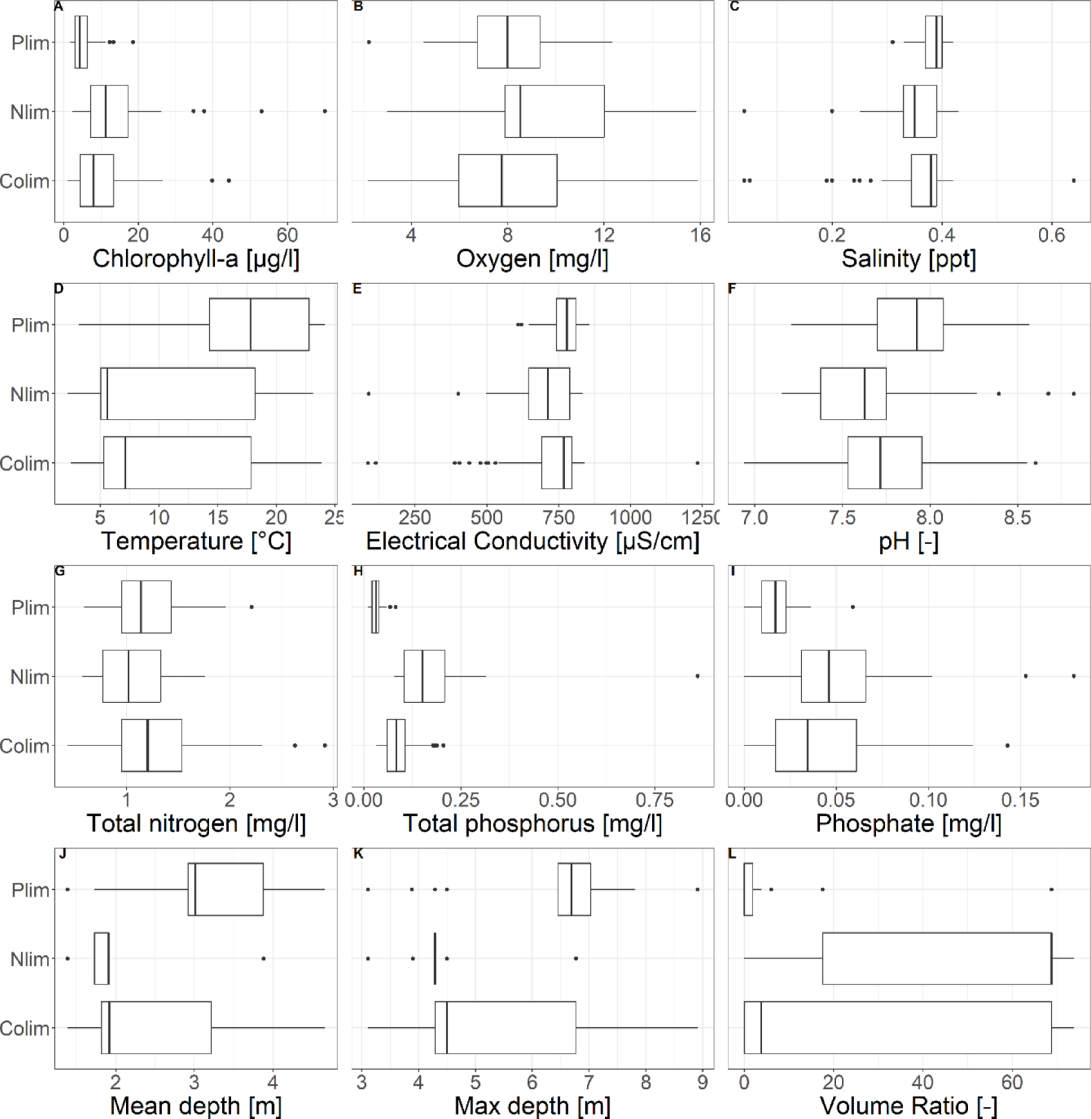
Distribution of measured parameters in sub-sets for three categories of nutrient limitation (Plim = P-limitation, Nlim = N-limitation, Colim = Co-limitation).

The limitation of nutrients for algae growth is mainly driven by the nutrient concentrations itself. To better understand the aquatic ecosystems of the Grunewald chain of lakes, the measured parameters, nutrient concentrations and the volume ratio as characteristic of the surrounding areas that affect the lakes were investigated concerning their distribution over the nutrient limitation categories (Fig. 5). The parameters that affect the target variable TN: TP ratio are grouped according to the categories P-limitation (Plim), N-limitation (Nlim) and Co-limitation (Colim) and summarized as box-plots. To give an example: with the concentration of total nitrogen (TN) and total phosphorus (TP) the TN: TP ratio is calculated for each sampling date and for each sampling site. According to the TN: TP ratio the chlorophyll-a concentration at the same sampling date and sampling site is categorized in one of the three categories. That means when the TN: TP ratio is higher than 23 on sampling date 1 at sampling site 1, the chlorophyll-a concentration of sampling date 1 and sampling site 1 is categorized as P-limited. This kind of categorization is done for all parameters.

In Fig. 5 the distribution of measured parameters for the three categories of nutrient limitation (P-limitation, N-limitation and Co-limitation) are shown. The following parameters do not vary strongly comparing the three categories: salinity and electrical conductivity. A tendency of lower values in P-limited lakes can be found for the parameters chlorophyll-a, total phosphorus, phosphate and volume ratio. Strongly higher values for P-limited lakes can be found for the parameters temperature, pH value, mean depth and maximum depth. The parameters oxygen and total nitrogen do not differ strongly in the three categories, but a tendency of higher oxygen concentration in N-limited and Co-limited lakes can be found as well as lower nitrogen concentrations in N-limited lakes. The results demonstrate distinct patterns in: lake depth, showing that deeper lakes tend to P-limitation while shallower lakes tend to Co- and N-limitation; temperature, which is higher in P-limited lakes; chlorophyll-a concentrations, which are higher in P-limited lakes; and phosphorus concentrations, that are lower in P-limited lakes and higher in Co- and N-limited lakes. These findings underscore how varying physical and chemical characteristics differently impact the ten lakes of the Grunewald Lake chain.

### Boruta analysis

**Fig. 6:**
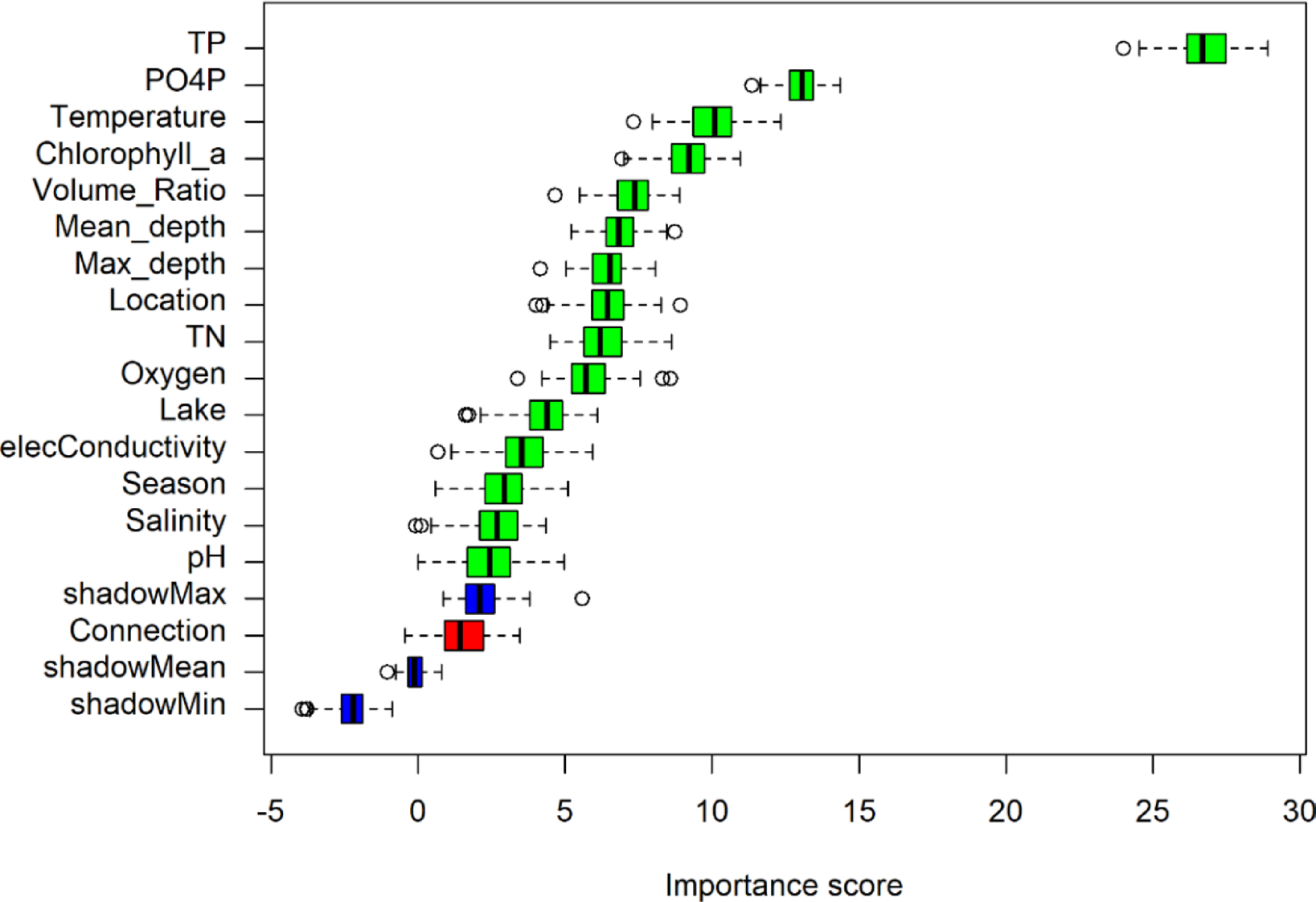
Boruta analysis showing the importance of parameters (y-axis) on the TN: TP ratio with blue boxplots for shadow, red as rejected and green as confirmed importance of parameters.

In the Boruta analysis (Fig. 6) total phosphorus has the highest importance score with a median value around 26 on the TN: TP ratio in the Grunewald chain of lakes. The importance score represents a range of values between 5 and 15 for the parameters ortho-phosphate, temperature, chlorophyll-a, volume ratio, the lake depths, total nitrogen and oxygen. Importance scores ranging from 3 to 5 are found for electrical conductivity, season, salinity and the pH-value. Importance scores below the maximum of the shadow are not important, which applies to the lake characteristic ‘type of connections between the lakes’. The ranking of the importance score provides information on which parameters should be prioritized to improve the TN: TP ratio.

## Discussion

Along the chain of lakes, the nutrient concentrations of total phosphorus, phosphate and total nitrogen are increasing from one lake to the next, yielding the highest nutrient loads in the last lake (Fennsee). Especially in urbanized catchments such as catchments from the lakes Dianasee to Fennsee, high phosphorus concentrations are found. With increasing phosphorus concentrations, the TN: TP ratio decreases along the chain of lakes, resulting in a shift from P-limited lakes to Co-limitation and N-limitation. The nutrient limitation in the Grunewald chain of lakes show relationships with nutrient loads, chlorophyll-a concentrations and lake characteristics such as lake depth. In the following sections, these findings are discussed separately. First, the pollution of the lakes is discussed. Secondly, the shift of the nutrient limitation along the chain of lakes is further analyzed. The discussion chapter ends with a deeper analysis on the parameters that affect the nutrient limitation in the Grunewald chain of lakes.

### Pollution of the lakes

The physical and chemical parameters underline the effect of sewage water from traffic areas in lakes that are more exposed to anthropogenic impact. The variation of electrical conductivity and salinity is larger for lakes with higher volume ratios (Hubertussee, Fennsee) and a catchment that is dominated by urban areas. The high amount of storm water runoff that flows in the Talgraben, the connection between the Hubertussee and Fennsee, affect both lakes after a heavy rainfall event in July 2025 which can be seen at the low electrical conductivity at stations 13 and 14. At station 17 the impact from the heavy rain event with water flowing from the streets through a lamella filter can be seen as well in July 2025. With rain water from traffic areas, soluble substances such as phosphate, salts and other organic substances can enter the lakes, cause eutrophication and increasing growth of algae. With an increase of algae growth (chlorophyll-a) there is also an increase of organic decomposition using oxygen leading to reduced oxygen concentrations. Low oxygen concentrations can impact the fixation of phosphorus on iron in the sediment as well as the denitrification and degradation of phosphorus^46^. Due to that, higher amounts of nutrients are not bound in the sediment, instead they stay in the water phase and increase the risk of eutrophication of lakes. In addition to the external nutrient inputs and the internal nutrient cycling, in the Grunewald chain of lakes the nutrient concentrations are transported from one lake to another. Whereas for total nitrogen a reduced concentration was found for the outflow of Schlachtensee, Krumme Lanke and Grunewaldsee. Starting from Dianasee, a higher risk of nutrient loads that are transported to the downstream lakes occurs. For total phosphorus and phosphate higher concentrations at the outflow of the lakes lead to an accumulation of high phosphorus concentrations in the last lakes Hubertussee and Fennsee. Considering the measured nutrients, especially the lake Fennsee is polluted by high amounts of nitrogen and phosphorus concentrations that affect the aquatic ecosystem. Polluted water that was transported to the lake Fennsee in the past led to an ecosystem with high nutrient loads stored in the system, but until now the Fennsee is fed with water from surrounding traffic areas that pollutes the ecosystems further in addition to nutrients that are transported along the chain of lakes. The water surface is covered by duck weed and a lot of hornworts can be identified below the water surface, which are common species in eutrophic systems^47,48^. The strong growth of water plants is supported by the high nutrient load in the lake. The seasonal shifts of the limiting nutrient are predominantly caused by reduced biological activity during the cold periods^49^ and ongoing high nutrient loads from surrounding areas in addition to the degradation of water plants and organic material^18,50^. Considering the physical and chemical parameters of the monitoring campaign, the Grunewald chain of lakes shows strongly decreasing water quality along the flow path. Nutrients are transported from one lake to another in addition to the high loads of sewage water to the urban lakes.

### The shift of the limiting nutrient

Due to reduced TN: TP ratios along the chain of lakes the nutrient limitation shows a shift from P-limitation to Co-limitation. Considering the measured concentrations of the nutrients, a decrease of nitrogen causes the shift to Co-limitation from lakes Krumme Lanke to Hundekehlesee. The concentration of total phosphorus is mainly stable and at low levels at these stations. Starting in the lake Dianasee, total phosphorus increases while total nitrogen stays stable, which causes the shift to a Co-limitation at the following lakes. Due to high total nitrogen concentrations in the lake Fennsee a N-limitation occurs during spring and winter.

The reduction of phosphorus at the surface water treatment plant Beelitzhof works well for the first three lakes of the Grunewald chain of lakes. These lakes are P-limited and further reduction of phosphorus is applicable to these lakes. According to Hilt et al.^12^ through the domino effect of connected lakes the last lake in a chain of lakes should profit from restoration measures as found in their study. At the Grunewald chain of lakes, the reverse of the flow direction improved the water quality of the first three lakes in 1981, but the following lakes from Dianasee to the Fennsee did not recover from the high nutrient loads in the past. The positive domino effect does not take place at the Grunewald chain of lakes as intended. Teurlincx et al.^7^ point out the cascading collapse of ecological states in connected water bodies which was found in their literature review on water quality of connected lakes. The reason for that might be the high amount of nutrients coming from the urbanized catchment into the lakes^7^. The high nutrient load from the surrounding urban area is also the main driver for the shift of the limiting nutrient for algae growth in the Grunewald chain of lakes. Higher concentrations of nitrogen cause a Co-limitation and at some stations (stations 12 to 17) a N-limitation. The variation of the TN: TP ratio and the limiting nutrient over time and over the seasons was also found by a study focusing on different lakes in England, where a shift of the TN: TP ratio was found over the seasons^51^. A reduction of both nutrients, nitrogen and phosphorus is likely to be more effective for urban lakes such as lakes of the Grunewald chain of lakes^21,38,52^. Most importantly, the inflowing nutrient loadings should be reduced. Moreover, it has to be considered that lake characteristics can influence the trophic state and nutrient limitation as well as the nutrient concentrations. In the study of McCullough et al.^16^ more than 3800 lakes in the United States were investigated considering their TN: TP ratio and the importance of parameters on the nutrient limitation. They found that the shift of the TN: TP ratio can also be caused by lake depth. These findings are complemented by the study of Qin et al.^15^ which shows that shallow lakes are more likely to be Co-limited and eutrophic, while deep lakes are more often P-limited. These findings are confirmed by our results. In the Grunewald chain of lakes, the first three lakes are deeper than the lakes Dianasee to Fennsee which are shallow lakes with mean depths around 2 m. The shallow lakes in the Grunewald chain of lakes are predominantly Co-limited and in some seasons N-limited, while the deeper lakes Schlachtensee to Grunewaldsee are P-limited. The shifts from P- to N-limitation were also found at other lakes in Berlin that were described as shallow and eutrophic^53^. The shift of the limiting nutrient was found as a general feature of lakes that is caused by seasonal changes in the rates of denitrification which is a major sink of nitrogen in lakes^54,55^, while the release of phosphorus from the sediment is described as an important internal source^46^. Moreover, in case of low oxygen concentrations at the sediment-water interface, the release of phosphorus from the sediment is increased^46^ as well as during higher temperatures in spring and summer that support the denitrification and the release of phosphorus^46^. The Grunewald chain of lakes, is dealing with several factors that affect the water quality and the nutrient limitation on algae growth. Upstream lakes are located in forested areas and have less inputs of urban areas compared to the downstream lakes that are surrounded by urban area and are used as storm water retention lakes. Moreover, downstream lakes are shallower than the upstream lakes, which make them more prone to shorter residence time and decreased degradation of nutrients. Most importantly, the chain of lakes is dealing with cascading effects with nutrient loadings that are transported from upstream to downstream lakes. To better understand the characteristics influencing the nutrient limitation in the Grunewald chain of lakes further analysis about the importance of parameters on the TN: TP ratio is conducted.

### Parameters affecting the TN:TP ratio

In the Grunewald chain of lakes chlorophyll-a is more dominant in lakes that are N-limited or Co-limited while P-limited lakes show a small amount of chlorophyll-a. Chlorophyll-a as indicator for phytoplankton and algae growth gives important information about the necessity of limiting phosphorus in lakes being more important than limiting nitrogen. In the study of Li et al.^56^ they analyzed the limiting nutrient in four constructed ponds where each pond receives a different amount of phosphorus, nitrogen and a combination of both to investigate the growth and community of phytoplankton as well as the limiting nutrient. They found out that the pond that received only phosphorus got the highest concentrations of chlorophyll-a. Their findings reveal that a limitation of phosphorus is necessary to control the phytoplankton growth. McCullough et al.^16^ revealed chlorophyll-a as the most important predictor for nutrient limitation in lakes, which was also found by Liang et al.^18^. In our study, chlorophyll-a does also have a high importance on the TN: TP ratio, but total phosphorus has a higher importance score on the TN: TP ratio in the Grunewald chain of lakes. Focusing on the nutrient concentrations in the Grunewald chain of lakes it becomes obvious that phosphorus plays a larger role in nutrient limitation than nitrogen. Total nitrogen concentrations vary to a less extent in the nutrient limitation categories whereas total phosphorus and ortho-phosphate are more damped in P-limited lakes than in N- and Co-limited lakes. Even though this behavior seems to be obvious, it is very important to consider that phosphorus plays a major role in the shift of TN: TP compared to nitrogen. These assumptions are complemented by the Boruta analysis which was conducted with the TN: TP ratio as target variable.

Considering the importance score of lake characteristics such as volume ratio and depth of the lake as well as the location which describes the sequence of the sampling stations along the flow path, the surrounding traffic areas and the discharge to the lakes affect the TN: TP ratio. Our study focuses on urban lakes and shows in addition to the study of McCullough et al.^16^ that other variables than chlorophyll-a such as lake depth and the concentrations of phosphate and total phosphorus can have a stronger importance on the limiting nutrient and also for the trophic state, than in studies where a high amount of different lakes that are not only affected by urban areas are investigated.

For the Grunewald chain of lakes a reduction of phosphorus to yield a P-limitation in the lakes is recommended, but still it should be considered for shallow water bodies that a reduction of both nutrients, phosphorus and nitrogen, can be applicable if the internal load of phosphorus is high^39,57–60^. The reduction of both nutrients were found relevant especially for eutrophic lakes^61^. Considering the phosphorus concentrations of the Grunewald chain of lakes investigated in our study, especially the lakes from Dianasee to Fennsee can be classified as eutrophic. In combination with their shallow morphology and the high load of nutrients from surrounding traffic areas a Co-limitation of phosphorus and nitrogen is recommended^62^.

## Conclusion

This study investigates the water quality of the Grunewald chain of lakes, consisting of ten urban lakes in Berlin, Germany. The Grunewald chain of lakes is affected by anthropogenic impacts due to storm water that flows from the surrounding urban and traffic areas through pipes and canals to the lakes where the nutrient and contaminant rich water is stored. Most of the aquatic ecosystems suffer from high nutrient loads which cause fish disease and an eutrophic state. Over a period of 13 months, the inlets, outlets and direct connections of the lakes were monitored by taking water samples that were analyzed according to the concentration of total phosphorus, ortho-phosphate and total nitrate in addition to the physical and chemical parameter temperature, electrical conductivity, salinity, pH-value, oxygen concentration and chlorophyll-a concentration that were obtained in the field. The monitoring data supports a better understanding of the exchange between the lakes and the limiting nutrient on algae growth. Increasing phosphorus concentrations were found along the chain of lakes which showed a cascading effect on the water quality, expressed as TN: TP ratio. Along the lake chain, the TN: TP ratio decreased and shifted from P-limitation in forest dominated lake catchments to Co-limitation and N-limitation in urbanized lake catchments. Analyzing the TN: TP ratio for the inlets, outlets and connections of the ten lakes showed P-limitation for deeper lakes and mainly Co-limitation and sometimes N-limitation for shallow lakes. This study reveals that the TN: TP ratio helps to understand the limiting nutrient in general, but more parameters should be considered for further management of the lakes. Phosphorus was the most important driver on the TN: TP ratio for all urban lakes as revealed in the Boruta analysis, in which the importance of physical and chemical parameters such as electrical conductivity or oxygen concentrations and lake characteristics such as depth or volume ratio on the TN: TP ratio were investigated. Even though phosphorus is meant to be the main threat to the aquatic ecosystem, for shallow, eutrophic lakes, a management of both nutrients, phosphorus and nitrogen, is recommended, especially the reduction of external inputs through drainage filter systems. Further research on the TN: TP ratio and further management of eutrophic lakes should consider the different characteristics of lakes such as lake depth and volume ratio. Future studies should focus on the nutrient sources in connected aquatic systems. In this study we show, how urban lakes are affected by high loads of nutrients and that even in connected systems, different management strategies should be considered for the individual situations of the lakes and their anthropogenic pressure. Furthermore, future research should emphasize the development of management strategies for urban lakes, including the treatment of stormwater runoff from traffic-areas and the implementation of in-lake measures to improve the biological and chemical degradation of nutrients.

## Data Availability

The datasets generated and/or analysed during the current study are available in the LeoPard repository of the University Library of the Technical University of Braunschweig, https://doi.org/10.24355/dbbs.084-202601261338-0.
